# Spirit of Soap as an Antiseptic

**Published:** 1899-07-29

**Authors:** 


### SPIRIT OF SOAP AS AN ANTISEPTIC.

We all know that spirit of wine is a good antiseptic, and that it is especially useful against the microbes concerned in the production of suppuration, and we most of us also know that its tendency to harden the skin rather than to remove the superficial layers of epidermis, makes it a somewhat unreliable antiseptic unless combined with a pretty free preliminary use of soap and water. Professor Mikulicz, of Breslau, has, however, hit upon the plan of combining the spirit with the soap and thus making one operation do. He makes a spirit of soap consisting of soft soap, alcohol, and water—practically our linimentum saponis, with the camphor and oil of rosemary left out. It has been found possible to disinfect the hands of the surgeon by simple washing in this spirit of soap, without using any water; and, after using this compound in his clinic for several months past, Professor Mikulicz is satisfied that the results are just as good as those which were formerly obtained by much more complex methods. The same compound has also been used for the disinfection of the skin of the patient. The stuff is harmless and free from odour, and does not roughen the hands as some antiseptic solutions do. In this it agrees with lysol, and, like it also, it leaves the hands rather slippery. This, however, can be overcome by wiping them on a piece of sterilised gauze, or by the use of the sterilised thread gloves which the professor has for some time past recommended. The process seems likely to be found convenient, and if further experience should show it to be effectual amid the ordinary surroundings of general practice, where alone there is now any serious difficulty in obtaining a reasonable, although, perhaps, not a theoretically perfect asepsis, we may look forward to its extensive employment.

